# Left atrial mitral valve cord: Unveiling complexity through advanced 3D TEE imaging - A case report

**DOI:** 10.1016/j.radcr.2024.07.167

**Published:** 2024-08-27

**Authors:** Mohammad Sahebjam, Amirhossein Yadegar, Mahkameh Farmanesh

**Affiliations:** a Department of Echocardiography, Tehran Heart Center, Cardiovascular diseases Research institute, Tehran University of Medical Sciences, Tehran, Iran; b Endocrinology and Metabolism Research Center (EMRC), Vali-Asr Hospital, Tehran University of Medical Sciences, Tehran, Iran; c Cardiology Department, Sayad Shirazi Hospital, Golestan University of Medical Sciences, Gorgan, Iran

**Keywords:** Mitral valve, Left atrium, 3-dimensional echocardiography, Left atrial mitral valve cord, Congenital abnormalities

## Abstract

This case report explores the left atrial mitral valve cord, an extremely rare congenital anomaly. Typically involving mitral valve leaflets and associated with mild mitral regurgitation, it is rarely documented independently. A 51-year-old patient presented with dizziness, and diagnostic challenges arose during transthoracic echocardiography (TTE). Advanced 3-dimensional transesophageal echocardiography (3D TEE) proved invaluable for accurate mapping, revealing a unique, unattached left atrial mitral valve cord.

## Introduction

The left atrial mitral valve cord, a rare congenital anomaly [1], has seldom been the focal point in existing literature. This anomaly is usually asymptomatic and discovered incidentally while evaluating other medical conditions .It typically involves the mitral valve leaflets and is associated with mild mitral regurgitation [2]. However, prior reports have documented only a few instances where the chord is unattached to the mitral valve, similar to our case [3]. Accurate identification and differentiation of intracardiac anomalies are essential parts of the diagnosis. Key differential considerations include thrombus, vegetation, tumor, chordae tendineae, and cor-triatriatum, each with distinct clinical characteristics and treatment modalities. Understanding these can enhance diagnostic accuracy and patient outcomes, ensuring appropriate clinical management and avoiding unnecessary procedures [4]. Additionally, understanding the left atrial mitral valve cord can be pivotal in guiding clinical decision-making, especially in scenarios where transcatheter techniques like mitral transcatheter edge-to-edge repair (MTEER) may be considered [5].

## Case report

A 51-year-old patient presented to our clinic with a chief complaint of dizziness. Vital signs and a thorough physical examination, including a neurological assessment, were unremarkable, and laboratory results were within the normal range. The electrocardiogram (ECG) showed a normal sinus rhythm and no ST-segment deviations.

Transthoracic echocardiography (TTE) revealed normal left ventricular function and wall motion. However, it presented a diagnostic challenge: a slender, mobile, echo-bright filamentous strand on the atrial aspect of the mitral valve, accompanied by mild mitral regurgitation (MR) but no signs of mitral stenosis (MS). Differential considerations included thrombus, vegetation, tumor, chordae tendineae, or cor-triatriatum.

**Fig. 1:**
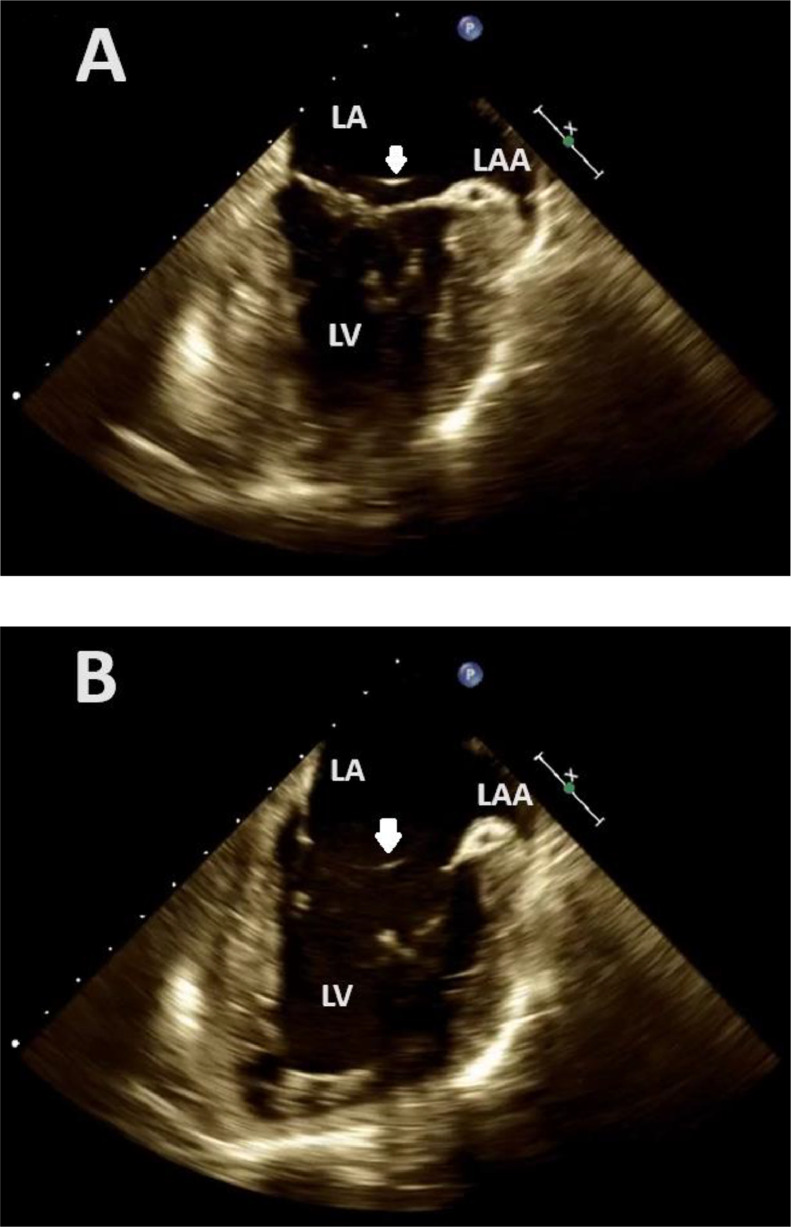
Two- and 3-dimensional transesophageal echocardiography showing a mid-esophageal commissural view during the systolic phase (A) and diastolic phase (B) demonstrate an echo dense structure (arrow) affixed to the left intra-atrial septum, extending across the mitral valve and connecting to the orifice of the left atrial appendage. LA, Left atrium; LV, Left ventricle; LAA, Left atrial appendage.

The subsequent 3-dimensional (3D) TEE provided a more in-depth characterization, revealing a slender, highly mobile, filamentous strand firmly attached to the septum of the left atrium with mild MR (Fig. 1, Fig. 2-Video 1, 2, 3, 4). The advanced 3D imaging proved invaluable in accurately mapping the chord's connection sites. This chord extended across the entire mitral valve, ultimately attaching to the inferior aspect of the left atrial appendage (LAA) orifice (Fig. 3-Video 5). Conclusive identification of the strand was achieved through cardiac imaging techniques such as TTE, transesophageal echocardiography (TEE), and 3-dimensional live imaging. These modalities enabled a comprehensive visualization of the strand's connection to the mitral valve [6]. The echogenicity of the chord was similar to that of the atrial wall and septum. Importantly, no evidence of thrombus or infection was observed in intracardiac chambers or on native valves. The advanced 3D TEE technology facilitated an unparalleled understanding of the complexities of this unique case, providing crucial insights for accurate diagnosis and treatment planning. The patient was discharged without intervention and reassurance was given.

**Fig. 2:**
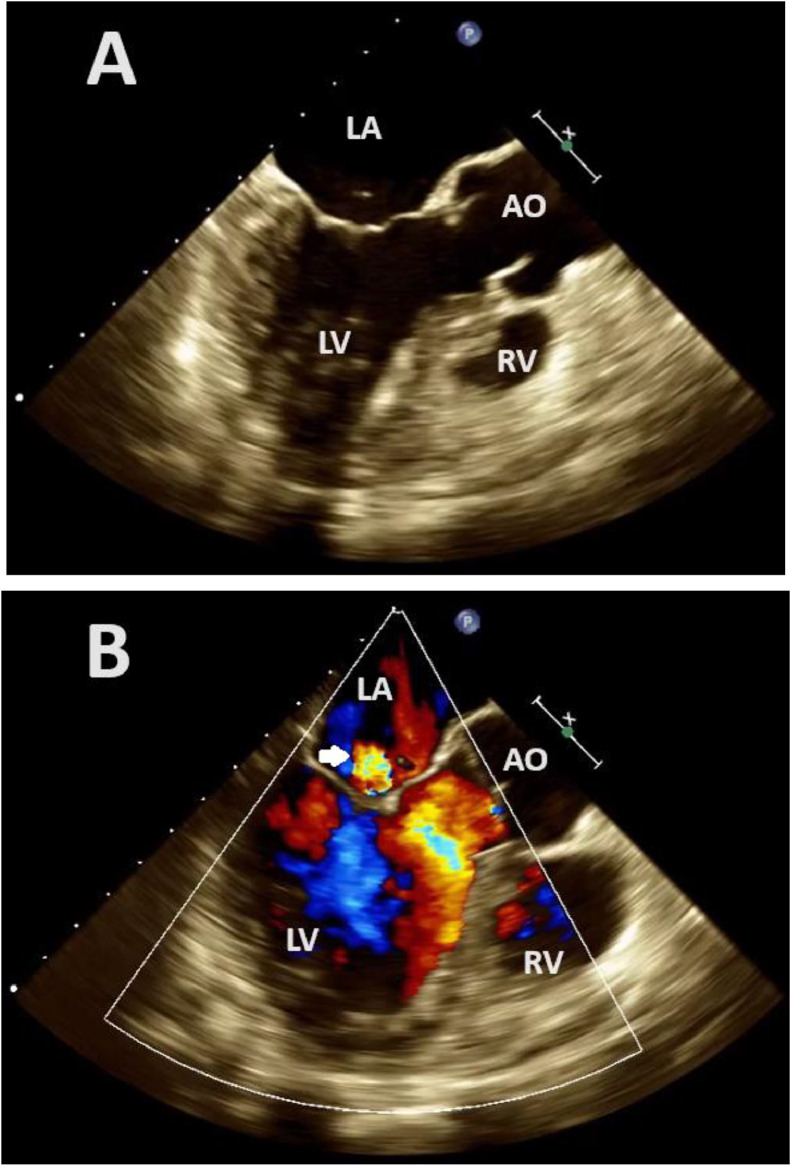
Two-dimensional transesophageal echocardiography at the mid-esophageal long-axis view during the systolic phase, without (A) and with (B) Color Flow Doppler, reveals mild mitral regurgitation (arrow). LA, the left atrium; LV, Left ventricle; RV, Right ventricle; AO, Aorta.

**Fig. 3:**
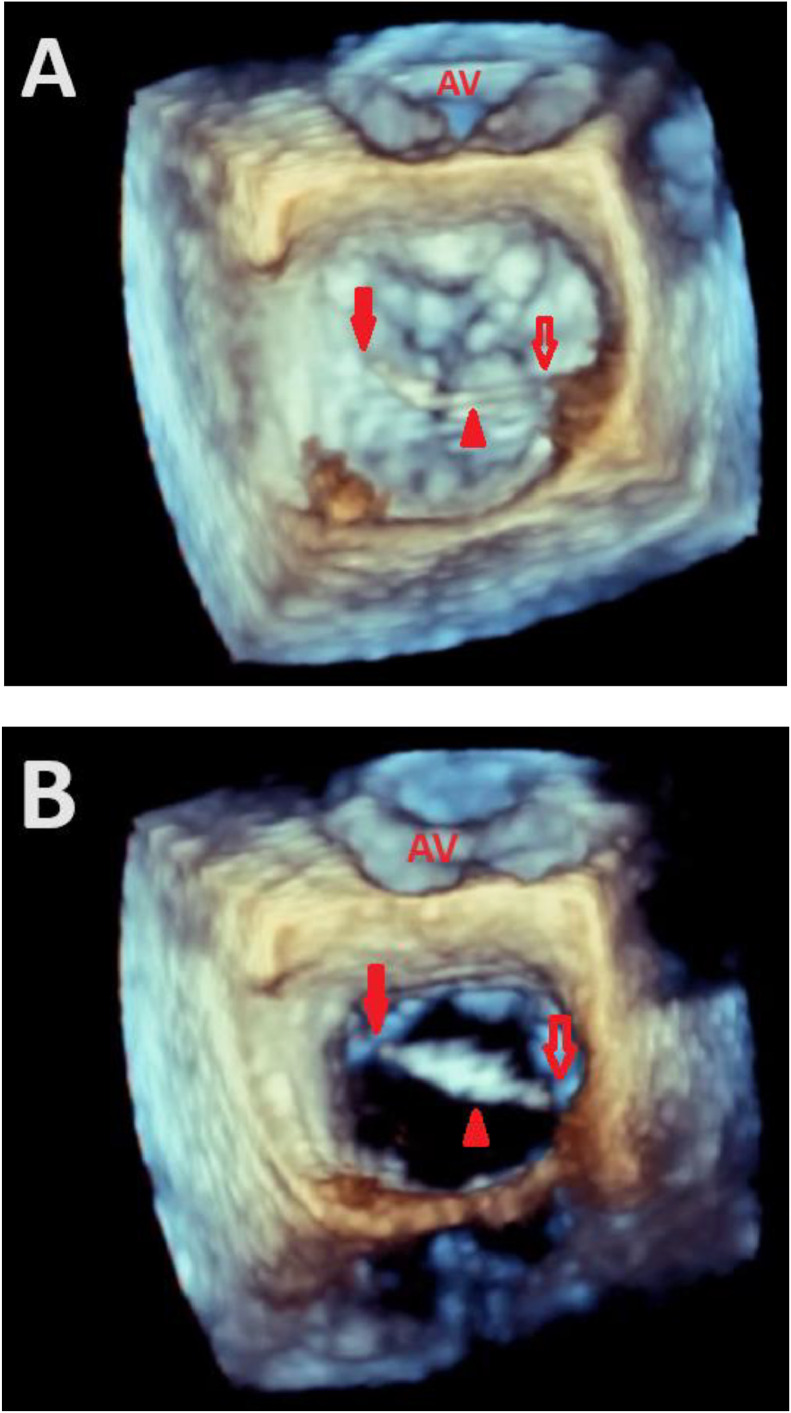
A 3-dimensional view of the mitral valve from the surgeon's perspective (with the aortic valve positioned at the top) during the systolic phase (A) and during the diastolic phase (B) highlight a left atrial chord (arrowhead) firmly attached to the interatrial septum (indicated by the open arrowhead) and connecting to the orifice of the left atrial appendage (as shown by the solid arrowhead). AV, Aortic valve.

## Discussion

The left atrial mitral valve cord is a rare congenital anomaly, with an estimated incidence of 2% in autopsy reports [1]. The prevalence and clinical relevance of these cords remain unclear, indicating a need for further research [7]. It is hypothesized that the chord associated with the left atrial mitral valve results from a developmental defect occurring between the 14th and 17th weeks of gestation, a period coinciding with the formation of papillary muscles and chordae during embryogenesis [8]. These bands are believed to be remnants of the septum primum, showing a leftward deviation and extension [3].

The left atrial mitral valve cord may present concurrently with other cardiac conditions, such as patent foramen ovale (PFO), Chiari's network, or premature atrial complexes, complicating its clinical presentation [9]. Individuals diagnosed with this anomaly range in age from 8 to 85 years, highlighting its relevance across all age groups [10,11]. Reported cases demonstrate a wide spectrum of presentations, from incidental findings with mild-to-severe MR [2,9,10] to isolated cases of infective endocarditis [12].

Previous cases have predominantly involved the anterior mitral valve leaflet (AMVL), especially the A2 scallop [2], with occasional reports implicating the posterior mitral valve leaflet (PMVL) [2,13]. This study aligns with a unique case where an anomalous chord was identified crossing the left atrium without direct contact with the mitral valve, presenting unusual manifestation of the left atrial mitral valve chord [3]. The case involved a 14-year-old boy who presented with vasovagal syncope, and subsequent evaluations also revealed a PFO [3].

Surgical intervention is recommended in cases with significant MR or infective endocarditis of the mitral valve [10,12]. However, asymptomatic patients typically require no further evaluation [4]. For patients with mild-to-moderate MR, serial echocardiograms are advised to monitor progression and provide valuable insights for clinical management [2].

The left atrial mitral valve cord is a rare, benign congenital anomaly with varied clinical presentations. Accurate recognition of this structure is crucial to avoid overdiagnosis and overtreatment. Additionally, in the era of trans-catheter edge-to-edge repair, the presence of a chord adds complexity to the diagnosis and treatment, highlighting the need for thorough understanding and precise identification to ensure effective patient management.

## Data availability

The data that support the findings of this study are included in the published article.

## Ethics approval

This study was conducted according to the declaration of Helsinki and was approved by the research ethics committee of the Tehran University of Medical Sciences.

## Author contributions

MS: Conceptualization, Writing-original draft preparation. AY: Data collection, Writing-review and editing. MF: Conceptualization, Data collection, Writing-original draft preparation. All authors read and approved the final manuscript.

## Patient consent

Written informed consent was obtained from the participant to publish her information without mentioning her name.

## Appendix Supplementary materials

Video 1: Two-dimensional TEE, mid-esophageal commissural view, reveals a mobile echo density attached to the lower part of the interatrial septum, extending over the mitral valve and connecting to the orifice of the left atrial appendage. Image, video 1

Video 2: Two-dimensional TEE, mid-esophageal long-axis view, depicts the mobile echo density attached to the intra-atrial septum, extending across the mitral valve. Image, video 2

Video 3: Two-dimensional TEE, mid-esophageal long-axis view, demonstrates mild mitral regurgitation without signs of mitral stenosis. Image, video 3

Video 4: Multi-plane imaging (x-plane) of mitral valve (MV) discloses a mobile echo density adherent to the lower aspect of the interatrial septum, extending across the mitral valve and connecting to the orifice of the left atrial appendage. Image, video 4

Video 5: Three-dimensional TEE of the mitral valve from the surgeon's perspective enhances the mapping of the chord's connection sites, showing attachment to the interatrial septum and connection to the left atrial appendage on the other side. Image, video 5
